# Low Infection Rate after Tumor Hip Arthroplasty for Metastatic Bone Disease in a Cohort Treated with Extended Antibiotic Prophylaxis

**DOI:** 10.1155/2015/428986

**Published:** 2015-02-01

**Authors:** Werner H. Hettwer, Peter Frederik Horstmann, Thea Bechmann Hovgaard, Tomas Andreas Grum-Scwensen, Michael M. Petersen

**Affiliations:** Musculoskeletal Tumor Section, Department of Orthopedic Surgery, Rigshospitalet, University of Copenhagen, 2100 Copenhagen, Denmark

## Abstract

*Background*. Compared to conventional hip arthroplasty, endoprosthetic reconstruction after tumor resection is associated with a substantially increased risk of periprosthetic joint infection (PJI), with reported rates of around 10% in a recent systematic review. The optimal duration of antibiotic prophylaxis for this patient population remains unknown. *Material and Methods*. To establish the infection rate associated with prolonged antibiotic prophylaxis in our department, we performed a retrospective review of all adult patients who underwent endoprosthetic reconstruction of the proximal femur after tumor resection for metastatic bone disease during a 4-year period from 2010 to 2013 (*n* = 105 patients). *Results*. Intravenous antibiotic prophylaxis was administrated for an extended duration of a mean of 7.4 days. The overall infection rate was 3.6% (4/111 implants), infection free survival was 96% at 2 years, and the risk of amputation associated with infection was 25% (1/4 patients). *Discussion*. Preemptive eradication of bacterial contamination may be of value in certain clinical situations, where the risk level and consequences of implant-associated infection are unacceptable. Our findings suggest that extended postoperative antibiotic prophylaxis may reduce the risk of PJI in patients undergoing tumor resection and endoprosthetic replacement for metastatic bone disease associated impending or de facto pathologic fractures of the proximal femur.

## 1. Introduction

Patients who undergo endoprosthetic reconstruction subsequent to malignant bone tumor resection are at high risk of prosthetic joint infection (PJI) (10-11%), as established in two large reviews [1, 2]. Although PJI is a devastating complication in itself, which may not only require further revision surgery, prolonged hospitalization, antibiotic treatment, and rehabilitation, it exposes the tumor patient in particular to significant further risks such as amputation [1–3] or compromised overall survival due to interference with radio- or chemotherapy. The substantial cost associated with treatment of PJI has also been well documented [4]. Prevention of this complication is of particular importance for patients undergoing tumor resection and endoprosthetic replacement for impending or de facto pathologic fractures secondary to metastatic bone disease, to preserve mobility and independence for as long as possible during the remainder of their lives and to spare them the drastic reduction in quality of life, invariably associated with PJI. As the optimal duration of antibiotic prophylaxis for this patient population is unknown, we wish to report our experience with extended postoperative intravenous antibiotic prophylaxis in these high-risk patients.

## 2. Materials and Methods

### 2.1. Study Design and Patient Population

**Figure 1:**
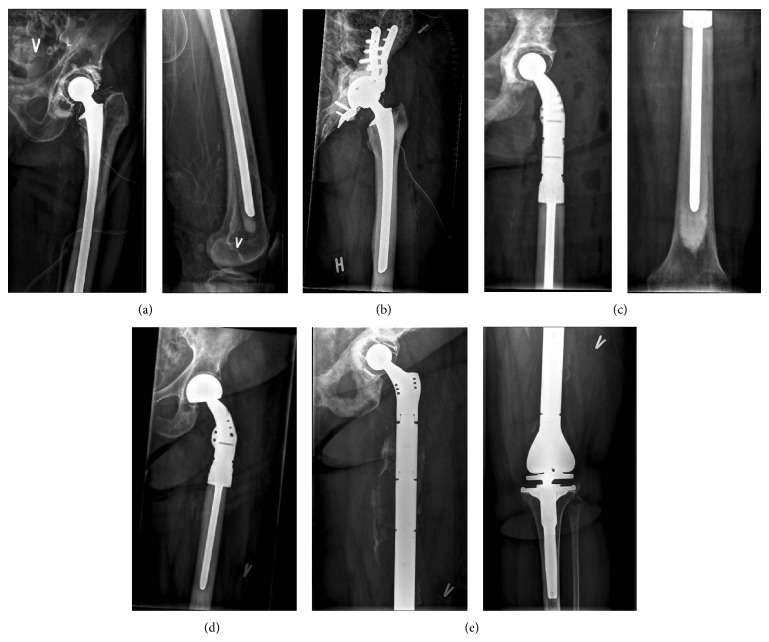
(a) Conventional neck resection, reconstructed with a full length (350 mm) cemented standard stem (Link SPII), to bridge metastatic involvement of the distal femur. Note screw fixation of an antiluxation device to the cemented polyethylene cup (Link Lubinus Eccentric). (b) Conventional calcar resection, reconstructed with a cemented standard stem (Link SPII, 200 mm). Note reconstruction and cement augmentation of a large concurrent acetabular lesion with a pelvic reconstruction cage (Link Partial Pelvic Replacement). (c) Proximal femur resection (160 mm), reconstructed with a cemented proximal femur replacement (Zimmer Segmental System) and a cemented acetabular component (Link Lubinus Eccentric). (d) Proximal femur resection (120 mm), reconstructed with a cemented proximal femur replacement (Zimmer Segmental System) and a multipolar femoral head. (e) Total femur replacement (Stryker GMRS). Note screw fixation of an antiluxation device to the cemented (Link Lubinus Eccentric) polyethylene cup and heterotopic bone formation around the diaphyseal part of the prosthesis.

We retrospectively reviewed the medical records of all patients with metastatic bone disease or malignant hematologic bone disease who underwent tumor resection and endoprosthetic reconstruction involving the proximal femur in our specialized orthopedic oncology unit between 2010 and 2013. We identified 105 patients (mean age = 65 (range 16–92) years, M/F = 45/60), who had received a total of 111 hip implants (Figures 1(a)–1(e)). The primary indication for surgery was de facto or impending pathological fracture of the proximal femur due to metastatic bone disease (Table 1). In 14 procedures (13%), substantial concurrent lesions of acetabulum or distal femur were present, requiring a more extensive surgical procedure, either acetabular reconstruction with a cemented partial pelvic replacement (*n* = 8), (Figure 1(b)) or total femur replacement (*n* = 6) (Figure 1(e)). All relevant demographic data (age, gender, nature and location of pathology, details of the surgical procedure, implants used, duration of antibiotic treatment, and hospital stay) were collected from the patient files. All postoperative emergency medical contacts or orthopedic hospital admissions, registered in our countrywide, national electronic medical record system, were reviewed to establish rate and type of relevant postoperative complications encountered. Complete data for patient survival and duration of hospital stay was available in all cases and for duration of antibiotic treatment in 107. Mean follow-up was 12.6 months (range 6 days–4.2 years), current as of September 1, 2014. The study was approved by the Danish Data Protection Agency (J.nr. 2013-41.2591). Ethical approval was not required in our country, as the present study was limited to review of medical records only and did not involve direct patient contact.

### 2.2. Surgical Procedure and Postoperative Routine

A routine posterior approach to the hip was employed in all patients. In 50 patients confinement of the tumor to the femoral head or neck allowed a conventional neck resection, preservation of the abductor mechanism, and endoprosthetic reconstruction with a cemented standard stem (Link SP2 (*n* = 24), Biomet Bi-Metric (*n* = 13), Implant Cast ic-Long Stem (*n* = 11), Implant Cast RS (*n* = 1), or Zimmer CPT (*n* = 1)) (Figure 1(a)). Fifty-five procedures required an extended posterior approach to accommodate the necessary proximal femoral resection (mean resection length 133 mm (range 50–325 mm)), and endoprosthetic reconstruction with either a cemented modular revision stem Link MP (*n* = 32) or a tumor megaprosthesis (Zimmer Segmental (*n* = 20), Link Mega C (*n* = 2), and Stryker GRMS (*n* = 1)) (Figures 1(c) and 1(d)). On the acetabular side, with exception of 14 hemiarthroplasties (Zimmer Multipolar), the majority of patients received a cemented acetabular component (Lubinus Eccentric, (*n* = 93)) or an uncemented cup (Zimmer Trilogy (*n* = 3), Biomet Ranawat (*n* = 1)). Eight cases required additional periacetabular tumor removal and pelvic reconstruction with a cemented pelvic reconstruction ring (Link Partial Pelvis Replacement (Figure 1(b)) and in 6 cases of metastatic disease involving the entire femur, total femur replacement (Link Mega C (*n* = 3), Stryker GRMS (*n* = 2), and Zimmer Segmental (*n* = 1)) was required (Figure 1(e)). All incisions were closed in a layered fashion over deep drains after detached musculature had been reattached to the prosthesis, in an attempt to restore the abductor mechanism and to cover the entire prosthesis with vital tissue. Postoperatively, all patients were mobilized, weight bearing as tolerated, from postoperative day 1. The sterile compressive dressing applied at conclusion of the procedure was left unchanged until day 2 or day 3 to coincide with removal of any drains still present. Intravenous antibiotics (Cefuroxime 1.5 g × 3) were started 30–60 min prior to incision and continued for 5 days and if necessary extended beyond 5 days until the wound was considered dry by a member of the surgical team. Thromboprophylaxis (Innohep 3500–4500 IE × 1 sc.) was maintained until the patients were well mobilized, at least until discharge from hospital. In eleven cases, after the surgical wound was dry, antibiotics had to be continued due to other causes: infection in the chest (*n* = 6), gastrointestinal tract (*n* = 2), urinary tract (*n* = 1), brain (*n* = 1), and one unknown primary focus.

### 2.3. Statistical Analysis

All data are presented as mean with total range, and *P* values of <0.05 were considered significant. We used standard IBM SPSS software (version 19) for the following statistical calculations: Kaplan-Meier survival analysis for estimation of overall patient survival and implant survival (time to infection, dislocation, and reoperation).

## 3. Results

### 3.1. Overall Survival

**Figure 2:**
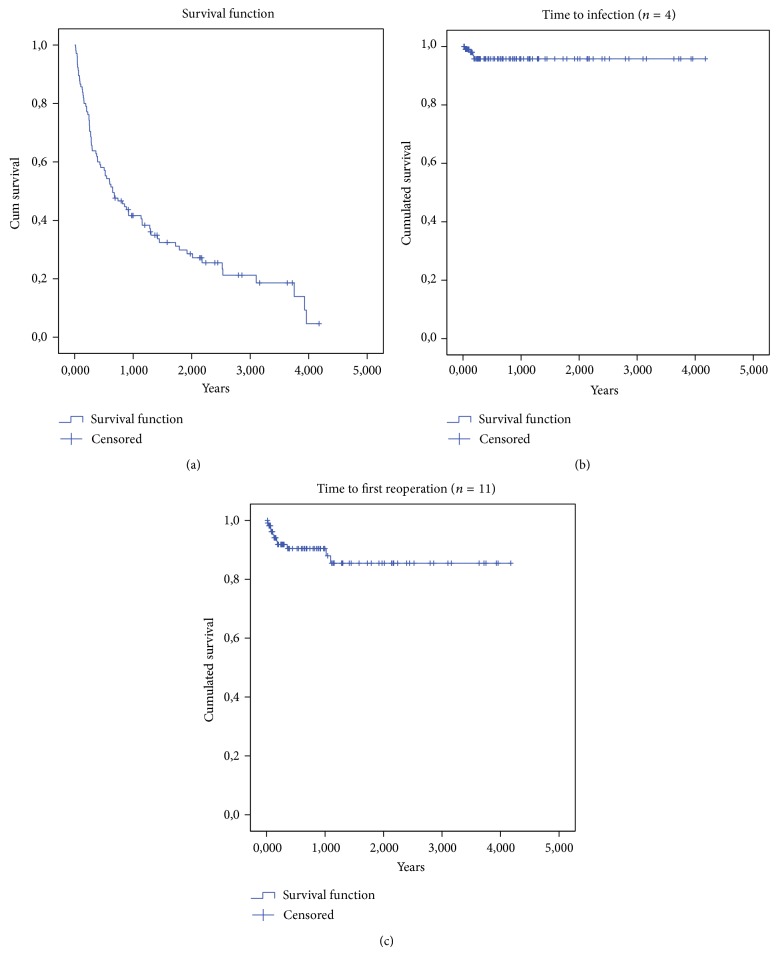
Kaplan Meier survival analysis showing cumulative survival rate (a), cumulative infection free survival rate (b), and cumulative reoperation free survival rate (c) for all patients (*n* = 105), who underwent endoprosthetic reconstruction of the hip for metastatic bone disease between 2010 and 2013.

As an indirect measure for the severity of the underlying metastatic bone disease, we performed an analysis of overall patient survival. Twelve patients died of their underlying disease within the first 30 postoperative days, mean survival was 12 months (range 0.2–50), and 26 patients remained alive (25%). The probability of overall survival was 74% at 3 months, 57% at 6 months, and 42% at 1 year (Figure 2(a)), which indicates that most patients were severely ill, with advanced stages of their underlying disease.

### 3.2. Duration of Antibiotic Treatment and Hospital Stay

The mean duration of treatment with antibiotics was 7.4 days (2–28 days), and the mean duration of hospital stay was 9.1 days (3–44 days).

### 3.3. Complications

We identified 16 complications (10 dislocations, 4 periprosthetic joint infections, one mechanical complication, and one local recurrence) resulting in a total of 15 revision procedures. Initial deep soft tissue debridement with exchange of mobile parts and implant retention was performed in all 4 cases of confirmed PJI. Two implants (in the same patient) could be retained with continuous oral antibiotic suppression for the remainder of her life, whereas rapid progression of infection around a total femur replacement, together with overall clinical deterioration, required exarticulation of the hip in another patient. Ongoing infection in the fourth case was managed with staged revision of the implant. One patient revised for progressive local recurrence and impending periprosthetic fracture remains on prophylactic oral antibiotic treatment due to positive cultures for propionibacteria, although this very likely represents a contamination (Table 2). Our overall infection rate was 3.6% (4/111 implants) with an associated risk of 25% for amputation (1/4 cases) and infection free survival of 96% at 3 months and 1 and 2 years (Figure 2(b)). The risk of reoperation for dislocation was 4% at 3, 6, and 12 months and 8% at 2 years, with a probability for dislocation free survival of 92% at 3 months, 91% at 6 months and 1 year, and 88% at 2 years, resulting in an overall probability for reoperation free survival of 92% at 3 months, 90% at 6 months and 1 year, and 86% at 2 years (Figure 2(c)).

## 4. Discussion

Extended antibiotic prophylaxis has been standard practice in our department for over 10 years and we have previously published a very low infection rate of only 2% in a study, evaluating all our patients who had received tumor joint replacements for metastatic bone disease from 2003 to 2008 [5]. However, as the countrywide electronic medical record system covering the entire population of our nation had not been established at that time, we could not be certain that some cases of PJI might have been missed. Now, as this is no longer the case, the observed infection rate of 3.6% (4/111 procedures) in this study still favorably compares with our previous results and remains substantially lower than those published in most other studies evaluating infection rates of tumor endoprostheses [1, 2]. Likewise, the observed 1-year survival rate of 42% corresponds well with our previous material from 2003 to 2008 [5], in which we found a 1-year survival rate of 39%. Larger, similar studies conducted by the Scandinavian Sarcoma Group found almost identical 1-year survival rates ranging from 39% in a study of 460 patients [6] to 41% in a study of 1195 patients [7]. Our 2-year infection free survival rate of 4% is probably slightly overestimated due to the well-known potential for bias of Kaplan Meier survival estimations in the presence of significant competing risks, such as death [8]. However, as the event of interest (infection) tended to occur very early, with all observed infections arising within the first 3 months and given the relatively short follow-up period, this overestimation is probably clinically insignificant. Excessively long perioperative antibiotic prophylaxis is generally regarded as an error by most experts in musculoskeletal infectious diseases; however, it is well recognized that certain conditions clearly benefit from preemptive therapy with administration of antibiotics over several days [9]. Just as treatment aimed at eradicating the often substantial bacterial wound contamination associated with high grade open fractures, prolonged administration of antibiotics may well have contributed to the comparatively low infection rate identified in our patients in a similar manner. Acknowledging their unique risk profile, potentially unacceptable adverse outcomes, and substantial costs associated with treatment of patients with an infected tumor joint replacement, most orthopedic oncologists feel strongly that standard guidelines for routine antibiotic prophylaxis should not be applied to patients with bone and soft-tissue tumors undergoing limb preserving endoprosthetic reconstructive surgery [10]. Despite the reportedly high infection rates of up to 25%, even higher reinfection rates as high as 43% [11], and the often dire consequences of PJI, resulting in amputation in up to 35% in some series [3], in an initiative led by the Musculoskeletal Infection Society and the European Bone and Joint Infection Society, a recent multinational consensus meeting on PJI recommended use of routine antibiotic prophylaxis, not exceeding 24 h, for patients undergoing major reconstruction with megaprosthesis [11], claiming lack of sufficient evidence that longer prophylaxis was warranted.

However, in a recent systematic review of 48 studies including a total of 4838 adult patients, Racano et al. could establish a clear trend towards long-term antibiotic prophylaxis being more effective in decreasing risk of PJI in patients undergoing tumor resection and endoprosthetic reconstruction of the lower limb, with pooled infection rates of only 8%, compared to pooled infection rates of 13% for antibiotic prophylaxis of 24 h or shorter [2]. Despite the relatively small, heterogeneous patient cohort and a retrospective design, our study supports this trend and favorably compares with the lowest infection rates reported in this systematic review. Given this very low infection rate identified in our patient cohort, we see no reason to change our current practice of extended antibiotic prophylaxis for these high-risk patients until very convincing evidence to the contrary becomes available. More definitive high level evidence to help resolve this controversy will probably not emerge for many years, as a randomized, controlled, and international multicenter trial, to determine the role of long-term antibiotics in patients undergoing surgical excision and endoprosthetic reconstruction for primary bone tumors, has only recently started enrolment of an anticipated total of 920 patients [12]. Unfortunately, by far the largest patient group undergoing tumor resection and endoprosthetic replacement, namely, those with impending or de facto pathologic fractures secondary to metastatic bone disease, will be excluded from this trial, so that the body of literature able to directly guide antibiotic prophylaxis in this particular patient group will remain very limited.

## 5. Conclusion

Preemptive eradication of bacterial contamination after endoprosthetic reconstruction subsequent to malignant bone tumor resection by means of extended postoperative antibiotic prophylaxis may reduce the risk of PJI in this high-risk patient population.

Further evidence is needed to confirm this hypothesis.

## Figures and Tables

**Table 1:** Demographics and pathology of all consecutive patients treated with tumor resection and endoprosthetic reconstruction of the hip between 2010 and 2013.

Number of patients	105
Female/male	60/45
Mean age at surgery (years)	65
(range)	(16–92)
Primary tumor	
Breast	35
Lung	19
Kidney	13
Prostate	10
Myeloma	8
Lymphoma	4
Colon	3
Esophagus	2
Bladder	2
Stomach	1
Anal	1
Oncocytoma	1
Planocellular	1
Gl. submandibularis	1
Uterus	1
Unknown	3
Indication for surgery	
Pathological fracture	70
Impending pathological fracture	41

**Table 2:** Cases with positive deep tissue cultures.

Number	Age (years)	Patient survival	Type of cancer	Implant	Time to infection	Microbiology	Outcome
1	80	1 yr. 4 m.	Lymphoma	Zimmer total femur	2,1 m.	*E. coli *	Hemipelvectomy
2	55	1 yr. 5 m.	Breast	Zimmer segmental	1,3 m.	*S. epidermidis *	Staged revision
3	38	2 yr. 6 m.	Breast	Link MP (right)	2,2 m.	*E. coli *	Implant retention and continuous suppressive antibiotic therapy
4	40	8 m.	Breast	Link MP (left)	0,4 m.	*S. aureus *	Implant retention and continuous suppressive antibiotic therapy
5	44	2 yr. 2 m.	Kidney	Link MP	13,2 m.	*P.acnes *	Revised for local recurrence, intraoperative cultures positive for *P. Acnes* (interpreted as likely contamination)

## References

[1] Jeys L. M., Grimer R. J., Carter S. R., Tillman R. M. (2005). Periprosthetic infection in patients treated for an orthopaedic oncological condition. *The Journal of Bone and Joint Surgery Series A*.

[2] Racano A., Pazionis T., Farrokhyar F., Deheshi B., Ghert M. (2013). High infection rate outcomes in long-bone tumor surgery with endoprosthetic reconstruction in adults: a systematic review. *Clinical Orthopaedics and Related Research*.

[3] Jeys L., Grimer R. (2009). The long-term risks of infection and amputation with limb salvage surgery using endoprostheses. *Recent Results in Cancer Research*.

[4] Hernández-Vaquero D., Fernández-Fairen M., Torres A. (2013). Treatment of periprosthetic infections: an economic analysis. *The Scientific World Journal*.

[5] Sorensen M. S., Gregersen K. G., Grum-Schwensen T., Hovgaard D., Petersen M. M. (2013). Patient and implant survival following joint replacement because of metastatic bone disease: a cross-sectional study of 130 patients with 140 joint replacements. *Acta Orthopaedica*.

[6] Hansen B. H., Keller J., Laitinen M. (2004). The Scandinavian Sarcoma Group Skeletal Metastasis Register. Survival after surgery for bone metastases in the pelvis and extremities. *Acta Orthopaedica Scandinavica, Supplement*.

[7] Ratasvuori M., Wedin R., Keller J. (2013). Insight opinion to surgically treated metastatic bone disease: scandinavian Sarcoma Group Skeletal Metastasis Registry report of 1195 operated skeletal metastasis. *Surgical Oncology*.

[8] Keurentjes J. C., Fiocco M., Schreurs B. W., Pijls B. G., Nouta K. A., Nelissen R. G. (2012). Revision surgery is overestimated in hip replacement. *Bone and Joint Research*.

[9] Swiss Orthopaedic Society (2014). *Infections of the Musculoskeletal System*.

[10] Musculoskeletal Tumor Society (2013). *Position Statement*.

[11] Musculoskeletal Infection Society http://www.msis-na.org/wp-content/themes/msis-temp/pdf/ism-periprosthetic-joint-information.pdf.

[12] Ghert M., Deheshi B., Holt G. (2012). Prophylactic antibiotic regimens in tumour surgery (PARITY): protocol for a multicentre randomised controlled study. *British Medical Journal*.

